# Hypertension Induced by Combination Therapy of Cancer: A Systematic Review and Meta-Analysis of Global Clinical Trials

**DOI:** 10.3389/fphar.2021.712995

**Published:** 2021-09-06

**Authors:** Xiaodan Guo, Xiaoyu Qian, Ying Jin, Xiangyi Kong, Zhihong Qi, Tie Cai, Lin Zhang, Caisheng Wu, Weihua Li

**Affiliations:** ^1^ Fujian Provincial Key Laboratory of Innovative Drug Target Research and State Key Laboratory of Cellular Stress Biology, School of Pharmaceutical Sciences, Xiamen University, Xiamen, China; ^2^ Department of Cardiology, Xiamen Key Laboratory of Cardiac Electrophysiology, Xiamen Institute of Cardiovascular Diseases, The First Affiliated Hospital of Xiamen University, School of Medicine, Xiamen University, Xiamen, China; ^3^ Department of Breast Surgical Oncology, National Cancer Center/National Clinical Research Center for Cancer/Cancer Hospital, Chinese Academy of Medical Sciences and Peking Union Medical College, Beijing, China; ^4^ Clinical Laboratory, Peking Union Medical College Hospital, Chinese Academy of Medical Science and Peking Union Medical College, Beijing, China; ^5^ State Key Laboratory of Coal Resources and Safe Mining, School of Chemical and Environmental Engineering, China University of Mining and Technology, Beijing, China; ^6^ School of Population Medicine and Public Health, Chinese Academy of Medical Sciences and Peking Union Medical College, Beijing, China; ^7^ Melbourne School of Population and Global Health, University of Melbourne, Melbourne, VIC, Australia

**Keywords:** hypertension, combination therapy, angiogenesis inhibitors, meta-analysis, randomized controlled trial

## Abstract

**Background:** Nowadays, due to the limitation of single therapy, combination therapy for cancer treatments has become important strategy. With the advancement of research on cardiotoxicities induced by anti-cancer treatment, among which cancer treatment-induced hypertension is the most frequent case. However, due to the small sample size and the absence of comparison (single-arm study alone), these studies have limitations to produce a feasible conclusion. Therefore, it is necessary to carry out a meta-analysis focusing on hypertension caused by cancer combination therapy.

**Methods:** We systematically searched PubMed, Embase, Cochrane Library, Web of Science, and CNKI, from database inception to November 31, 2020, with randomized controlled trials (RCTs) associated with hypertension induced by cancer combination drugs. The main endpoint of which was to assess the difference in the incidence of hypertension in cancer patients with monotherapy or combination therapy. We calculated the corresponding 95% confidence interval (95% CIs) according to the random effect model and evaluated the heterogeneity between different groups.

**Results:** According to the preset specific inclusion and exclusion criteria, a total of 23 eligible RCTs have been included in the present meta-analysis, including 6,241 patients (Among them, 2872 patients were the control group and 3369 patients were the experimental group). The results showed that cancer patients with combination therapy led to a higher risk of hypertension (All-grade: RR 2.85, 95% CI 2.52∼3.22; 1∼2 grade: RR 2.43, 95% CI 2.10∼2.81; 3∼4 grade: RR 4.37, 95% CI 3.33∼5.72). Furthermore, compared with the control group who received or did not receive a placebo, there was a higher risk of grade 3-4 hypertension caused by cancer combination treatment.

**Conclusion:** The present meta-analysis carries out a comprehensive analysis on the risk of patients suffering from hypertension in the process of multiple cancer combination therapies. Findings in our study support that the risk of hypertension may increase significantly in cancer patients with multiple cancer combination therapies. The outcomes of this meta-analysis may provide a reference value for clinical practice and may supply insights in reducing the incidence of hypertension caused by cancer combined treatment.

## Introduction

Hypertension has been recognized as the most common comorbidity among various types of cancers, which directly affects the prognosis of cancer patients, and is one of the high-risk factors for cancer survivors suffering from the comorbidity of heart diseases (Jain and Townsend, 2007). In the early stage of diagnosis, there is generally a similar probability of developing hypertension. However, with different cancer treatment patterns, patients may experience significantly altered incidence of hypertension, especially those receiving chemotherapy, which can reach 38% (Piccirillo et al., 2004; Maitland et al., 2010). In addition, novel cancer therapies, such as targeted therapy, which is a type of cancer treatment that targets proteins controlling cancer cells’ growth, division, and spreading, are also associated with the incidence of hypertension. Cardio-Oncology is an evolving discipline which aims to analyze the relationship between cancer treatment and cardiotoxicity (Lenneman et al., 2016; Barac, 2020). Cardiovascular toxicity in cancer treatment refers to the occurrence of cardiovascular disease during the disturbance or elimination of cancer cells in patients *in vivo*. Significantly, cardiovascular disease is the second leading cause of the morbidity and mortality of cancer survivors. According to previous studies, the probability of all-grade hypertension is between 15 and 67% during the treatment by using small molecule vascular endothelial growth factor tyrosinase inhibitors (e.g., sunitinib, sorafenib, pazopanib, etc.), and the rate would be higher with the use of inhibitors with higher efficiency (e.g., axitinib) (Brinda et al., 2016). The incidence of hypertension induced by tyrosinase inhibitors ranges from 5 to 80% in a dose-dependent manner (Agarwal et al., 2018). In addition, some patients may have a history of hypertension before the diagnosis of cancer. However, some patients develop hypertension due to anti-cancer treatment, and hypertension may be the direct result of cancer treatment under this circumstance.

The progress of cancer treatment has promoted the development of multiple new treatment strategies. Combination therapies means combining two or more therapies for cancer patients and the effectiveness may be excellent than single therapy. However, most programs will be accompanied by a series of cardiovascular adverse reactions, especially the existed high correlation of some new drugs with hypertension. In addition, the use of some chemotherapy drugs can also induce hypertension.

Generally, angiogenesis is a necessary process of tumorigenesis, growth, and metastasis. Vascular endothelial growth factor (VEGF) is an angiogenic growth factor. Angiogenesis inhibitor is a classic drug highly associated with the occurrence of hypertension (Hamnvik et al., 2015), primarily including monoclonal antibodies and small-molecule drugs. It has been documented that the proposed highly specific drugs are important inhibitors of angiogenesis, which play a role by blocking the signaling pathways necessary for angiogenesis, such as blocking Vascular Endothelial Growth Factor Receptor (VEGFR), Epidermal Growth Factor Receptor (EGFR), basic Fibroblast Growth Factor (bFGF), Platelet-derived Growth Factor Receptor (PDGFR), etc. (Folkman, 2007). To be specific, VEGF is the main growth factor that controls angiogenesis. Epidermal growth factor (EGF) is responsible for differentiation and apoptosis. bFGF can regulate the proliferation and differentiation of specific types of cells and has an effective effect on angiogenesis. Platelet-derived growth factor (PDGF) involves significantly cell growth, cell division, and angiogenesis (Wilkins et al., 2014; Agarwal et al., 2018).

With the emergence of various novel approaches to cancer treatment, the survival of cancer patients is becoming higher, which, however, is accompanied by an increasingly more obvious change in cardiotoxicity. Given the differences in cancer tissue types, therapeutic drugs, and drug doses, a systematic review and meta-analysis were carried out on hypertension caused by cancer treatment (Said et al., 2017), which aimed to clarify the incidence and risk of hypertension in cancer patients treated with combination therapy. At present, there is incomplete knowledge of hypertension caused by cancer combination therapy. Besides, there is few systematic reviews or meta-analyses in this aspect based on the comprehensive analysis of previous literature. Accordingly, through comprehensive literature analysis, it is expected to analyze and elaborate the risk factors of hypertension caused by cancer combination therapy, to provide a certain reference value for clinical treatment.

## Methods

The present systematic review and meta-analysis were conducted following PRISMA guidelines (Moher et al., 2009). The protocol has been registered in PROSPERO with the registration number CRD42021220923.

### Data Sources and Searches

A comprehensive literature search was made in databases [PubMed, embase, Cochrane Library, Web of Science, and CNKI] since November 31, 2020, to identify all articles related to the subject. In addition to the above databases, the clinical trial registration website (https://clinicaltrials.gov/) was searched to obtain information about registered prospective trials.

The keywords used in PubMed were listed as follows:

1) randomized controlled trial [pt]
2) controlled clinical trial [pt]
3) randomized [tiab]
4) placebo [tiab]
5) clinical trials as topic [mesh: noexp]
6) randomly [tiab]
7) trial [ti]
8) (1) OR (2) OR (3) OR (4) OR (5) OR (6) OR (7)
9) animals [mh] NOT humans [mh]
10) (8) NOT (9)

The final selected literatures were checked and reviewed separately to include the latest and most complete clinical trial reports in the case of repeated publications. All the search results were incorporated into the management tool of Endnote.

### Study Selection and Data Extraction

The major objective of our study was to determine the incidence of hypertension associated with combination therapy for cancer and to establish a relationship between combination therapy and the risk of hypertension. Therefore, eligible studies were those evaluating the combination of drugs with hypertension induced in cancer patients. Phase I trial was excluded considering the multi-dose level and limited sample size. In addition, phase II, III, and IV randomized controlled trials (RCTs) in combination therapy were enrolled in the analysis compared with those without combination therapy.

The eligible studies met the inclusion criteria:

1) Phase II, III, and IV trials involving cancer patients;
2) RCTs for cancer treatment;
3) Intervention group: combination therapy (including targeted therapy and chemotherapy);
4) Control group: monotherapy or placebo treatment;
5) Studies with available data on hypertension events or incidence and sample size.

The exclusion criteria:

1) Review articles
2) Not randomized control trial
3) Reports from same study sample
4) Not report associate with hypertension
5) Not report associate with cancer combination therapy
6) No usable data
7) No comparable trial
8) Republished literature

Two investigators (G.X and Q.X) extracted data independently, and any disagreements between the two reviewers were resolved by consensus. Online studies before publication were also eligible, but not including reviews, Conference reviews, studies published only in abstract form, quality of life research, non-randomized trials, and studies that could not determine the toxicity of combination therapy. Data extraction covered author, year of publication, research institution, journal name, trial phase, cancer tissue type, combination therapy, number of patients, age of patients, administration schedule and drug dose, size of control group, number of patients with hypertension, with the data of hypertension at all grades extracted.

### Data Synthesis and Analysis

Statistical analysis of this study was performed by using the Cochrane Review Manager (RevMan 5.3) software provided by the Cochrane Library Collaboration Network.

The proportion of patients with hypertension in each study was calculated by dividing the number of patients with hypertension caused by combination therapy extracted from eligible clinical trials by the total number of patients receiving combination therapy in each study. We refer to all levels of hypertension events as “All-grade,” “1–2 grade” is combined the grade of 1 or 2 hypertension events, and “3–4 grade” which is the sum of the level of 3 or 4 hypertension events.

For each study enrolled in this analysis, the relative risk (RR) and 95% confidence interval (95% CI) of the incidence of events between the intervention group and the control group were calculated according to the number of reported events and sample size. The *I*2 index and Q-statistics were used to evaluate the heterogeneity among studies, among which the Q-test is widely used at present (Zintzaras and Ioannidis, 2005). *p* < 0.05 of the Q-test indicated the existence of heterogeneity (Zhang et al., 2019), and *p* < 0.05 meant the existence of statistical significance. If *p* > 0.05, the results of the independent studies might be homogeneous, suggesting the use of the fixed-effect model; On the contrary, the random-effect model should be used and/or consider the clinical suitability of combination therapy when there was heterogeneity with *p* < 0.05. *I* ^2^ can quantify the heterogeneity among studies, which is calculated generally based on χ^2^ test. It describes the percentage of variation among studies in total variation, which may indicate a higher heterogeneity with the increase of the value of *I* ^2^ (Huedo-Medina et al., 2006). *I* ^2^ > 25, 50, and 75% suggest that there may be low, moderate, and high heterogeneity among studies. Besides, it is generally believed that there is substantial heterogeneity when *I* ^2^ > 50%.

## Results

### Search Results

**FIGURE 1:**
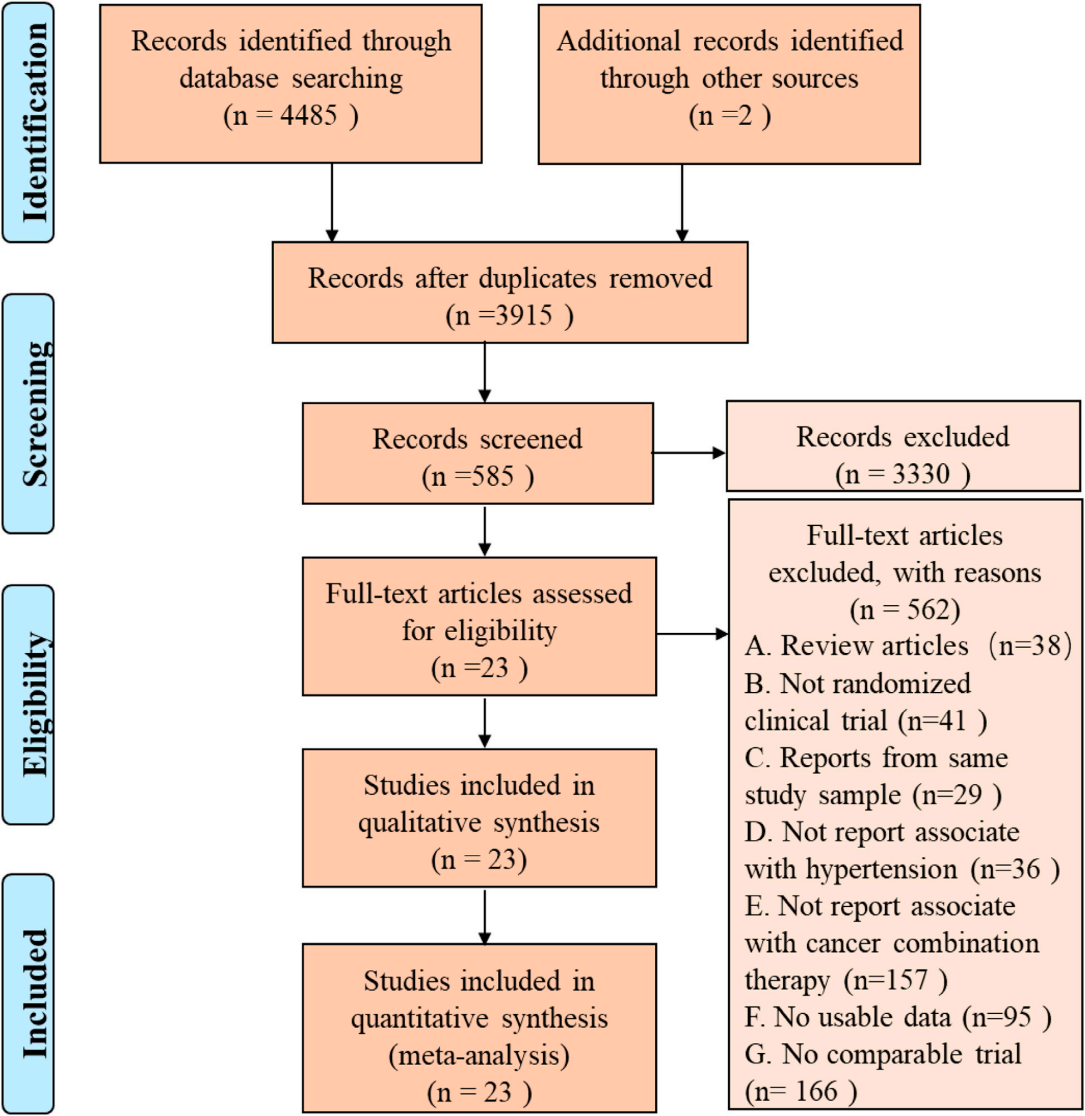
PRISMA Flow Diagram of Study Selection for this Meta-Analysis.

A total of 3,915 articles were identified by literature search and reference list review. After screening and qualification evaluation, 23 clinical trials involving 6,241 patients were finally included after excluding review articles, case reports, and meta-analysis articles, with the flow chart of literature selection shown in Figure 1. Of the 23 studies, there were 12 phase II, 11 phase III, and 1 phase IV trials, with the year of publication ranging from 2005 to 2020 (Table 1) (Miller et al., 2005; Heymach et al., 2008; Goss et al., 2010; Mok et al., 2011; Rugo et al., 2011; Baselga et al., 2012; Kato et al., 2012; Johnston et al., 2013; Laurie et al., 2014; Liu et al., 2014; Mackey et al., 2015; Rini et al., 2016; Baselga et al., 2017; Kubota et al., 2017; Yan et al., 2017; Dummer et al., 2018; Lu et al., 2018; Liu et al., 2019; Nakagawa et al., 2019; Cortot et al., 2020; Guo et al., 2020; Sinn et al., 2020; Tao et al., 2020). According to the published Common Terminology Criteria for Adverse Events (CTCAE) by the National Cancer Institute (NCI), hypertension caused by anti-cancer treatment includes 5 grades of grade 1–5 (Table 2) (National Cancer Institute, 2017). Among them, grade 5 hypertension includes fatal elevated blood pressure. There were no patients with grade 5 hypertension in the included literatures. Consequently, only grade 1–4 hypertension was enrolled in the data extraction. After research, there is no discovery showing that the patients enrolled in the reviewed RCTs were taking anti-hypertensive drugs.

**TABLE 1:** Characteristics of the studies included in this meta-analysis.

Entry	Author	Year	Country	Institution	Journal	Study phase	Cancer type	Combination therapy	Intervention arm information					Control arm information					Ref
									Patient number	Age (Range)	Hypertension Event		Regimen	Patient number	Age (Range)	Hypertension Event		Regimen	
											Grade 1–2	Grade 3–4				Grade 1–2	Grade 3–4		
1	Miller et al.(2005)	2005	United States	Indiana University	Journal of Clinical Oncology	III	Breast Cancer	Capecitabine + bevacizumab vs Capecitabine	232	29–78	13	41	Orally Capecitabine (2,500 mg/m^2^/d) twice daily for 14 days followed by a 7-days rest period, bevacizumab (15 mg/kg) intravenously on day 1 of each 3-weeks cycle. Patients continued therapy for a maximum of 35 cycles	230	30–77	4	1	Orally Capecitabine (2,500 mg/m^2^/d) twice daily for 14 days followed by a 7-days rest period. Patients continued therapy for a maximum of 35 cycles	16
2	Heymach et al.(2008)	2008	United States	Dana-Farber Cancer Institute	Journal of Clinical Oncology	II	Non-Small-Cell Lung Cancer	Vandetanib + Paclitaxel and Carboplatin vs Placebo + Paclitaxel and Carboplatin	56	36–79	14	4	Orally vandetanib (300 mg) + Paclitaxel (200 mg/m^2^) and Carboplatin (area under the concentration-time curve at steady-state, 6 mg/ml· min) once every 3 weeks for a maximum of six cycles	52	42–83	2	0	Orally Placebo + Paclitaxel (200 mg/m^2^) and Carboplatin (area under the concentration-time curve at steady-state, 6 mg/ml· min) once every 3 weeks for a maximum of six cycles	17
3	Goss et al.(2010)	2010	Canada	The Ottawa Hospital Cancer Centre	Journal of Clinical Oncology	II/III	Non-Small-Cell Lung Cancer	Cediranib + Paclitaxel and Carboplatin vs Placebo + Paclitaxel and Carboplatin	126	36–77	19	19	Paclitaxel 200 mg/m^2^ by intravenous 3-h infusion and carboplatin dosed to an area under the serum concentration-time curve of 6 every 3 weeks for 6 to 8 cycles, cediranib 30 mg was administered orally once daily concurrently with chemotherapy	125	39–81	8	2	Paclitaxel 200 mg/m^2^ by intravenous 3-h infusion and carboplatin dosed to an area under the serum concentration-time curve of 6 every 3 weeks for 6 to 8 cycles, placebo was administered orally once daily concurrently with chemotherapy	18
4	Mok et al.(2011)	2011	China	Prince of Wales Hospital	Asia-Pacific Journal of Clinical Oncology	III	Non-Small-Cell Lung Cancer	Bevacizumab + Cisplatin and Gemcitabine vs Placebo + Cisplatin and Gemcitabine	34	35–79	16	3	Bevacizumab 15 mg/kg plus Cisplatin was administered i.v. at 80 mg/m^2^on day 1 and gemcitabine was administered i.v. at 1,250 mg/m^2^ on days 1 and 8. Chemotherapy every 3 weeks for up to six cycles	33	29–75	5	0	Placebo + Cisplatin was administered i.v. at 80 mg/m^2^on day 1 and gemcitabine was administered i.v. at 1,250 mg/m^2^ on days 1 and 8. Chemotherapy every 3 weeks for up to six cycles	19
5	Rugo et al.(2011)	2011	United States	University of California	Journal of Clinical Oncology	II	Breast Cancer	Docetaxel + axitinib vs Docetaxel + Placebo	112	30–79	26	5	Docetaxel 80 mg/m^2^ once every 3 weeks plus axitinib 5 mg twice per day	56	34–71	0	0	Docetaxel 80 mg/m^2^ once every 3 weeks plus placebo twice per day	20
6	Baselga et al.(2012)	2012	United States	Massachusetts General Hospital Cancer Center	Journal of Clinical Oncology	II	Breast Cancer	Capecitabine + Sorafenib vsCapecitabine + Placebo	115	55 (mean)	17	1	Capecitabine 1,000 mg/m^2^ orally twice a day for days 1–14 of every 21-days cycle with sorafenib 400 mg orally twice a day	114	54(mean)	10	2	Capecitabine 1,000 mg/m^2^ orally twice a day for days 1–14 of every 21-days cycle with placebo orally twice a day	21
7	Kato et al.(2012)	2012	Japan	National Hospital Organization Osaka National Hospital	Annals of Oncology	II	Colorectal Cancer	Cediranib + mFOLFOX6 vs Placebo + mFOLFOX6	58	33–79	43	4	Once-daily cediranib 20 mg combination with 14-days treatment cycles of mFOLFOX6 (oxaliplatin 85 mg/m^2^ IV, day 1; leucovorin 200 mg/m^2^ IV, day 1; 5-FU 400 mg/m^2^ IV bolus, day 1 and then 2400 mg/m^2^ continuous IV infusion over 46 h)	58	36–80	17	1	Once-daily placebo combination with 14-days treatment cycles of mFOLFOX6 (oxaliplatin 85 mg/m^2^ IV, day 1; leucovorin 200 mg/m^2^ IV, day 1; 5-FU 400 mg/m^2^ IV bolus, day 1 and then 2,400 mg/m^2^ continuous IV infusion over 46 h)	22
8	Kato et al.(2012)	2012	Japan	National Hospital Organization Osaka National Hospital	Annals of Oncology	II	Colorectal Cancer	Cediranib + mFOLFOX6 vs Placebo + mFOLFOX6	56	40–82	42	6	Once-daily cediranib 30 mg combination with 14-days treatment cycles of mFOLFOX6 (oxaliplatin 85 mg/m^2^ IV, day 1; leucovorin 200 mg/m^2^ IV, day 1; 5-FU 400 mg/m^2^ IV bolus, day 1 and then 2,400 mg/m^2^ continuous IV infusion over 46 h)	58	36–80	17	1	Once-daily placebo combination with 14-days treatment cycles of mFOLFOX6 (oxaliplatin 85 mg/m^2^ IV, day 1; leucovorin 200 mg/m^2^ IV, day 1; 5-FU 400 mg/m^2^ IV bolus, day 1 and then 2,400 mg/m^2^ continuous IV infusion over 46 h)	22
9	Johnston et al.(2013)	2013	United Kingdom	Institute of Cancer Research	Breast Cancer Research and Treatment	II	Breast Cancer	Lapatinib + pazopanib vs Lapatinib	36	33–82	12	2	Daily lapatinib 1,500 mg plus pazopanib 800 mg	72	29–80	3	0	Daily lapatinib 1,500 mg	23
10	Laurie et al.(2014)	2014	Canada	University of Ottawa	European Journal of Cancer	II/III	Non-Small-Cell Lung Cancer	Cediranib + Paclitaxel and Carboplatin vs Placebo + Paclitaxel and Carboplatin	153	23–85	36	15	Paclitaxel (200 mg/m^2^) and carboplatin (area under the concentration time curve 6) intravenously every 3 weeks. Daily oral cediranib 20 mg was commenced day 1 of cycle 1 and continued as monotherapy after completion of 4–6 cycles of chemotherapy	153	36–77	11	3	Paclitaxel (200 mg/m^2^) and carboplatin (area under the concentration time curve 6) intravenously every 3 weeks. Daily oral placebo was commenced day 1 of cycle 1 and continued as monotherapy after completion of 4–6 cycles of chemotherapy	24
11	Liu et al.(2014)	2014	United States	Dana-Farber Cancer Institute	Lancet Oncol	II	Ovarian Cancer	Cediranib + Olaparib vs Olaparib	44	32–82	17	18	Cediranib 30 mg daily and olaparib capsules 200 mg twice daily	46	42–86	0	0	Olaparib capsules 400 mg twice daily	25
12	Mackey et al.(2015)	2015	Canada	Cross Cancer Institute	Journal of Clinical Oncology	III	Breast Cancer	Ramucirumab + Docetaxel vs Placebo + Docetaxel	759	24–82	152	51	Docetaxel 75 mg/m^2^ plus ramucirumab 10 mg/kg once every 3 weeks	385	29–81	37	7	Docetaxel 75 mg/m^2^ plus placebo once every 3 weeks	26
13	Rini et al.(2016)	2016	United States	Cleveland Clinic Taussig Cancer Institute	The Lancet Oncology	II	Renal Cell Carcinoma	IMA901 + sunitinib vs Sunitinib	204	56–69	27	24	Sunitinib (50 mg) was given orally once daily with each cycle defined as 4 weeks on treatment followed by 2 weeks off treatment, plus up to ten intradermal vaccinations of IMA901 (4·13 mg) and granulocyte macrophage colony-stimulating factor (75 μg) and with one dose of cyclophosphamide (300 mg/m^2^) 3 days before the first vaccination	135	54–66	24	7	Sunitinib (50 mg) was given orally once daily, with each cycle defined as 4 weeks on treatment followed by 2 weeks off treatment	27
14	Baselga et al.(2017)	2017	United States	Memorial Sloan Kettering Cancer Center	Clin Breast Cancer	III	Breast Cancer	Sorafenib + Capecitabine vs Placebo + Capecitabine	266	53 (Median)	32	36	Capecitabine (1,000 mg/m^2^ bid on days 1–14 of each 21-days cycle) plus sorafenib (600 mg/day)	271	55 (Median)	9	6	Capecitabine (1,000 mg/m^2^ bid on days 1–14 of each 21-days cycle) plus placebo	28
15	Kubota et al.(2017)	2017	Japan	Graduate School of Medicine	Journal of Clinical Oncology	III	Non-Small-Cell Lung Cancer	Motesanib + Paclitaxel and Carboplatin vs Placebo + Paclitaxel and Carboplatin	197	59–70	54	32	Once daily oral motesanib 125 mg and received paclitaxel 200 mg/m^2^ IV and carboplatin area under the concentration-time curve 6 mg/ml·min IV on day 1 of each 3-weeks cycle for up to six cycles	204	58–69	25	4	Once daily oral placebo and received paclitaxel 200 mg/m^2^ IV and carboplatin area under the concentration-time curve 6 mg/ml·min IV on day 1 of each 3-weeks cycle for up to six cycles	29
16	Yan et al.(2017)	2017	China	Baoji Central Hospital	Cancer Research and Clinic	Ⅳ	Gastric Cancer	Apapatinib + Oxaliplatin and Tiggio vs Oxaliplatin and Tiggio	75	34–75	17	0	Apatinib 850 mg/d, 0.5 h after meal begin oral administration, from the first day of chemotherapy and each 4-weeks cycle. Oxaliplatin (130 mg/m^2^ bid on 1 day of each 21-days cycle^)^ IV. Tiggio depends on the body surface area (<1.25m^2^ take 40 mg,1.25m^2^–1.50m^2^ take 50 mg, > 1.50m^2^ take 60 mg, twice a day)	75	34–75	0	0	Oxaliplatin (130 mg/m^2^ bid on 1 day of each 21-days cycle) IV. Tiggio depends on the body surface area (<1.25m^2^ take 40 mg,1.25m^2^–1.50m^2^ take 50 mg, > 1.50m^2^ take 60 mg, twice a day)	30
17	Dummer et al.(2018)	2018	Switzerland	University Hospital Zürich Skin Cancer Center	Lancet Oncol	III	Melanoma	Encorafenib + binimetinib vs Encorafenib	192	20–89	16	12	Encorafenib 450 mg once daily orally plus binimetinib 45 mg twice daily orally	194	23–88	5	6	Encorafenib 300 mg once daily orally	31
18	Lu et al.(2018)	2018	China	Jiao Tong University	Journal of Clinical Oncology	II	Non-Small-Cell Lung Cancer	Fruquintinib + Best supportive care vs Placebo + Best supportive care	61	54 (Median)	9	5	Oral fruquintinib (5 mg once daily) was given in 4-weeks cycles of 3 weeks of treatment followed by 1 week off, and combination with best supportive care	30	55 (Median)	0	1	Oral placebo was given in 4-weeks cycles of 3 weeks of treatment followed by 1 week off, and combination with best supportive care	32
19	Liu et al.(2019)	2019	United States	Dana-Farber Cancer Institute	Annals of Oncology	II	Ovarian Cancer	Cediranib + Olaparib vs Olaparib	44	-	16	18	Cediranib 30 mg daily and olaparib capsules 200 mg twice daily	46	-	0	0	Olaparib capsules 400 mg twice daily	33
20	Nakagawa et al.(2019)	2019	Japan	Kindai University Faculty of Medicine	The Lancet Oncology	III	Non-Small-Cell Lung Cancer	Ramucirumab + erlotinib vs Placebo + erlotinib	224	57–71	48	52	Oral erlotinib (150 mg/day) plus intravenous ramucirumab (10 mg/kg) once every 2 weeks	225	56–70	15	12	Oral erlotinib (150 mg/day) plus placebo once every 2 weeks	34
21	Cortot et al.(2020)	2020	France	Thoracic Oncology Department	European Journal of Cancer	III	Non-Small-Cell Lung Cancer	Paclitaxel + bevacizumab vs	111	18–81	14	8	90 mg/m2of paclitaxel (D1, D8, D15) plus 10 mg/kg of bevacizumab (D1,D15) every 28 days	55	35–78	0	0	Docetaxel (75 mg/m2) every 21 days	35
	Docetaxel																		
22	Guo et al.(2020)	2020	China	Shandong Provincial Hospital Affiliated to Shandong University	Medicine (Baltimore)	II	Cervical Cancer	Apatinib + Paclitaxel and Carboplatin vs	30	28–62	10	2	500 mg apatinib mesylate orally in between chemotherapy cycles, 135–175 mg/m^2^ paclitaxel (diluted in 500 ml of 0.9% saline and infused intravenously over 3 h) on day 1 and carboplatin AUC 5 (diluted in 500 ml of 0.9% saline solution and infused intravenously over 30 min) on day 2 every 3 weeks, for 6 cycles	29	30–69	2	0	135–175 mg/m^2^ paclitaxel (diluted in 500 ml of 0.9% saline and infused intravenously over 3 h) on day 1 and carboplatin AUC 5 (diluted in 500 ml of 0.9% saline solution and infused intravenously over 30 min) on day 2 every 3 weeks, for 6 cycles	36
								Paclitaxel and Carboplatin											
23	Sinn et al(2020)	2020	Germany	Department of Medical Oncology and Hematology	European Journal of Cancer	II	Pancreatic Cancer	Sorafenib + Gemcitabine	57	38–78	-	3	The average weekly dose of gemcitabine was 690 mg/m^2^, the average daily dose of sorafenib in the GemSorafenib arm was 650 mg (planned 800 mg daily)	65	43–80	-	1	The average weekly dose of gemcitabine was 690 mg/m^2^ and placebo	37
								Vs											
								Placebo + Gemcitabine											
24	Tao et al.(2020)	2020	China	Medicine School of University of Electronic Science and Technology	Dose-Response	III	Cervical Cancer	Paclitaxel + Carboplatin + bevacizumab vs	127	30–70	21	11	Intravenous 175 mg/m^2^ paclitaxel, intravenous 6 mg/mL/min area under the curve carboplatin, and intravenous 15 mg/m^2^ bevacizumab (Roche, Holding AG) every 3 weeks	161	30–70	11	5	Intravenous 175 mg/m^2^ paclitaxel (Taxol; Bristol-Myers Squibb) and intravenous 6 mg/mL/min area under the curve carboplatin (Paraplatin; Bristol-Myers Squibb) every 3 weeks	38
								Paclitaxel + Carboplatin											

**TABLE 2:** Characterized of hypertension in CTCAE.

Hypertension				
Grade 1	Grade 2	Grade 3	Grade 4	Grade 5
Adult: Systolic BP 120–139 mm Hg or diastolic BP 80–89 mm Hg	Adult: Systolic BP 140–159 mm Hg or diastolic BP 90–99 mm Hg if previously WNL; change in baseline medical intervention indicated; recurrent or persistent (≥24 h); symptomatic increase by > 20 mm Hg (diastolic) or to >140/90 mm Hg; monotherapy indicated initiated	Adult: Systolic BP≥160 mm Hg or diastolic BP≥100 mm Hg; medical intervention indicated; more than one drug or more intensive therapy than previously used indicated	Adult and Pediatric: Life-threatening consequences (e.g., malignant hypertension, transient or permanent neurologic deficit, hypertensive crisis); urgent intervention indicated	Death
Pediatric: Systolic/diastolic BP > 90th percentile but< 95th percentile	Pediatric and adolescent: Recurrent or persistent (≥24 h) BP > ULN; monotherapy indicated; systolic and/or diastolic BP between the 95th percentile and 5 mmHg above the 99th percentile	Pediatric and adolescent: Systolic and/or diastolic >5 mmHg above the 99th percentile		
Adolescent: BP ≥ 120/80 even if < 95th percentile	Adolescent: Systolic between 130 and 139 or diastolic between 80 and 89 even if < 95th percentile			

In this study, cancer types were Breast Cancer (*n* = 6), Cervical Cancer (*n* = 2), Colorectal Cancer (*n* = 1), Gastric Cancer (*n* = 1), Melanoma (*n* = 1), Non-Small-Cell Lung Cancer (*n* = 8), Ovarian Cancer (*n* = 2), Pancreatic Cancer (*n* = 1), and Renal Cell Carcinoma (*n* = 1). As for cancer combination therapy regimens, there was the combination of 2 drugs (*n* = 14), 3 drugs (*n* = 8), and >3 drugs (*n* = 1). Among the 23 therapeutic regimens, there were targeted therapy combined with chemotherapy (*n* = 17), two targeted therapies combined with chemotherapy (*n* = 5), and targeted therapy combined with other treatments (*n* = 1). In the control group, 10 studies adopted monotherapy, and 13 studies used placebo combined with monotherapy.

In all eligible studies, the average age of patients ranged from 18 to 89 years old. Among the eligible research articles, papers published in the United States accounted for the majority, with 8 articles, followed by China with 5 articles, Canada with 3 articles, Japan with 3 articles, Britain with 1 article, France with 1 article, Germany with 1 article and Switzerland with 1 article. Meanwhile, 8 articles were published in “Journal of Clinical Oncology,”, 4 in “The Lancet Oncology,”, and 3 in “European Journal of Cancer.”

### Evaluation of Included Studies

The Modified Jadad Scores scale (Jadad et al., 1996) was used to evaluate the quality of the 23 eligible articles. Following the evaluation based on the Randomization, Concealment of Allocation, Double Blinding, Withdrawals, and Dropouts, etc., there were 15 articles in 7 points, 5 articles in 5 points, 3 articles in 4 points, and 1 article in 3 points, as shown in Table 3.

**TABLE 3:** Scoring of modified Jadad measuring scale of the included studies.

Author (Year)		Randomization	Concealment of allocation	Double blinding	Withdrawals and dropouts	Score
Baselga et al. (2012)		2	2	2	1	7
Baselga et al. (2017)		1	1	2	1	5
Cortot et al. (2020)		2	1	1	1	5
Dummer et al. (2018)		2	2	2	1	7
Goss et al. (2010)		2	2	0	1	5
Guo et al. (2020)		1	1	0	1	3
Heymach et al. (2008)		1	1	2	1	5
Johnston et al. (2013)		0	2	1	1	4
Kato et al. (2012)		2	2	2	1	7
Kato et al. (2012)		2	2	2	1	7
Kubota et al. (2017)		2	2	2	1	7
Laurie et al. (2014)		1	2	0	1	4
Liu et al. (2014)		2	2	2	1	7
Liu et al. (2019)		2	2	2	1	7
Lu et al. (2018)		2	2	2	1	7
Mackey et al. (2015)		2	2	2	1	7
Miller et al. (2005)		2	2	2	1	7
Mok et al. (2011)		1	1	2	1	5
Nakagawa et al. (2019)		2	2	2	1	7
Rini et al. (2016)		2	2	2	1	7
Rugo et al. (2011)		2	2	2	1	7
Sinn et al. (2020)		2	2	2	1	7
Tao et al. (2020)		2	2	2	1	7
Yan et al. (2017)		1	1	1	1	4

### Relative Risk of Hypertension

**FIGURE 2:**
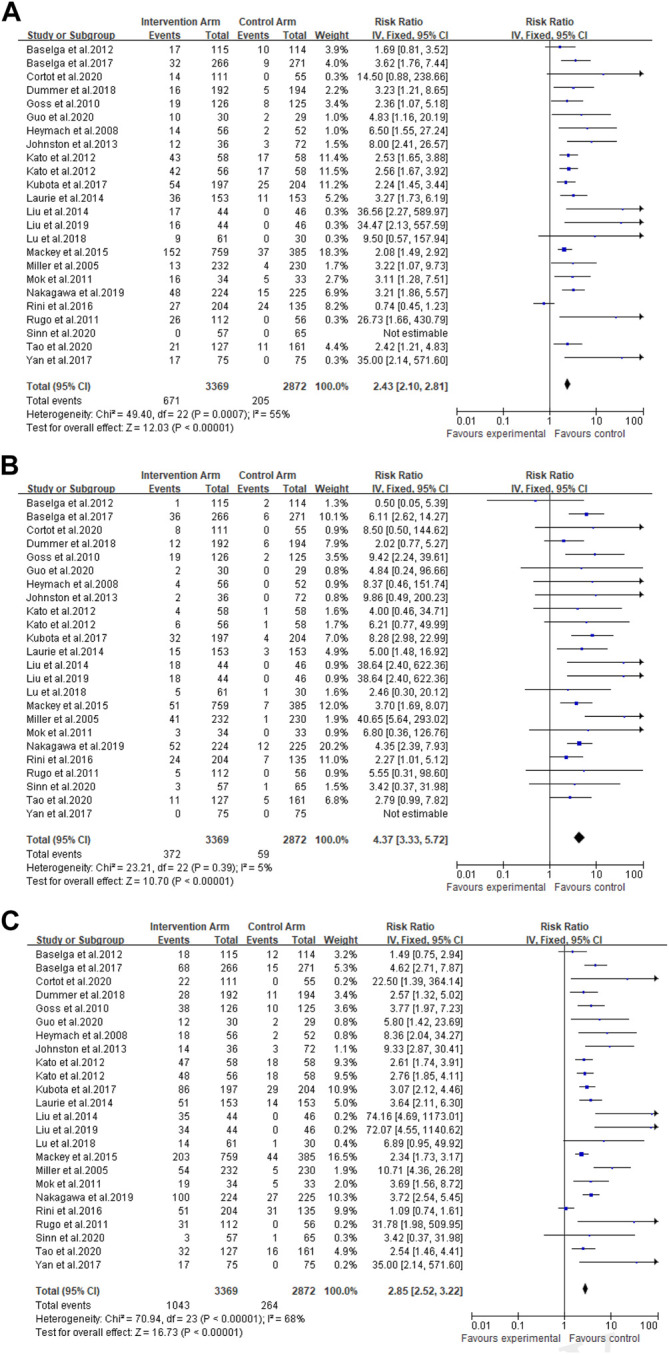
**(A)** Forest Plots for the Overall Comparison of Hypertension caused by cancer combination therapy. **(B)** Summary Relative Risks for Hypertension at Grade 3–4. **(C)** Summary Relative Risks for Hypertension at All Grade.

A total of 3,369 patients received cancer combination therapy, as well as 2,872 patients received cancer single therapy and/or placebo, which was available for comparative analysis. The incidence of grade 1–2 hypertension events ranged from 0 to 75%, and cediranib combined with mFOLFOX6 for the treatment of Colorectal Cancer had the highest probability of inducing hypertension (Kato et al., 2012). However, no events were observed in grade 1–2 hypertension in one trial (Sinn et al., 2020). Using the random-effect model, the RR in all patients developing grade 1–2 hypertension was 2.43 [95% CI 2.10–2.81, *p* < 0.001, Figure 2A]. Furthermore, the probability of grade 3–4 hypertension in all patients ranged from 0 to 40.9%, among which cediranib combined with Olaparib in treating Ovarian Cancer showed the highest probability of developing hypertension events (Liu et al., 2014; Liu et al., 2019). However, no grade 3–4 hypertension events were observed in the use of Oxaliplatin combined with oxaliplatin and Tiggio in the treatment of Gastric Cancer (Yan et al., 2017). Based on the random-effect model, the RR in all patients developing grade 3–4 hypertension was 4.37 [95% CI 3.33–5.72, *p* < 0.001, Figure 2B]. In addition, the incidence of all-grade hypertension ranged from 5.26 to 85.71%, and the highest incidence of hypertension was observed in the use of cediranib combined with mFOLFOX6 for the treatment of Colorectal Cancer (Kato et al., 2012). In the random-effect model, the RR in all patients developing grade 3–4 hypertension was 2.85 [95% CI 2.52–3.22, *p* < 0.001, Figure 2C].

### Overall Comparison of Hypertension

For all grades of hypertension, cancer patients receiving combination therapy had a relatively higher probability of developing hypertension (All-grade: RR 2.85, 95% CI 2.52–3.22; 1–2 grade: RR 2.43, 95% CI 2.10–2.81; 3–4 grade: RR 4.37, 95% CI 3.33–5.72) (Figure 2). In terms of all grades of hypertension caused by targeted drugs combined with chemotherapy, schemes with a relatively higher risk of developing hypertension included Paclitaxel combined with bevacizumab (RR 22.50, 95%CI 1.39–364.14) (Cortot et al., 2020), cediranib combined with Olaparib (RR 74.16, 95%CI 4.69–1,173.01; RR 72.07, 95%CI 4.55–1,140.62) (Liu et al., 2014; Liu et al., 2019), Docetaxel combined with axitinib (RR 31.78, 95%CI 1.98–509.95) (Johnston et al., 2013), as well as apapatinib combined with Oxaliplatin and Tiggio (RR 35.00, 95%CI 2.14–571.60) (Yan et al., 2017).

**FIGURE 3:**
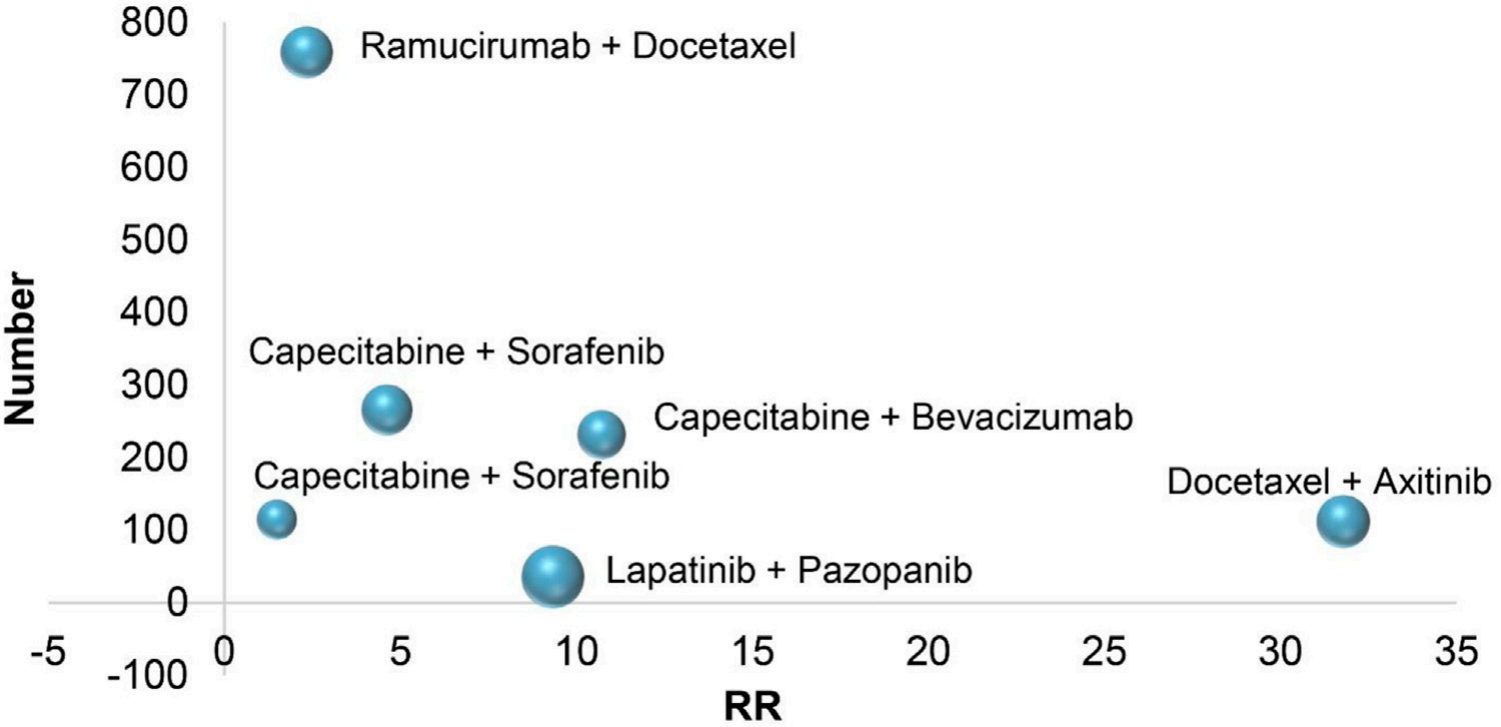
Bubble chart: Breast cancer treatment.

In six RCTs on the treatment of breast cancer, combination therapies included Capecitabine combined with bevacizumab (All-grade: RR 10.71, 95% CI 4.36–26.28; 1–2 grade: RR 3.22, 95% CI 1.07–9.73; 3–4 grade: RR 40.65, 95% CI 5.64–293.02) (Miller et al., 2005), Docetaxel combined with axitinib (All-grade: RR 31.78, 95% CI 1.98–509.95; 1–2 grade: RR 26.73, 95% CI 1.66–430.79; 3–4 grade: RR 5.55, 95% CI 0.31–98.60) (Rugo et al., 2011), Capecitabine combined with Sorafenib (All-grade: RR 1.49, 95% CI 0.75–2.94; 1–2 grade: RR 1.69, 95% CI 0.81–3.52; 3–4 grade: RR 0.50, 95% CI 0.05–5.39) (Baselga et al., 2012), lapatinib combined with pazopanib (All-grade: RR 9.33, 95% CI 2.87–30.41; 1–2 grade: RR 8.00, 95% CI 2.41–26.57; 3–4 grade: RR 9.86, 95% CI 0.49–200.23) (Johnston et al., 2013), ramucirumab combined with Docetaxel (All-grade: RR 2.34, 95% CI 1.73–3.17; 1–2 grade: RR 2.08, 95% CI 1.49–2.92; 3–4 grade: RR 3.70, 95% CI 1.69–8.07) (Mackey et al., 2015), Sorafenib combined with Capecitabine (All-grade: RR 4.62, 95% CI 2.71–7.87; 1–2 grade: RR 3.62, 95% CI 1.76–7.44; 3–4 grade: RR 6.11, 95% CI 2.62–14.27) (Baselga et al., 2017). According to the treatment of breast cancer, the RR of combination therapies induced hypertension is different, and the RR of Docetaxel combined with axitinib is higher than that of other treatments. In the combined treatment of breast cancer patients, Figure 3, it is not difficult to see that the RR of hypertension caused by ramucirumab combined with Docetaxel is small when the number of patients is gradually increasing, which indicates that ramucirumab combined with Docetaxel is the best treatment for low risk of hypertension caused by breast cancer in 6 RCTs of this research.

**FIGURE 4:**
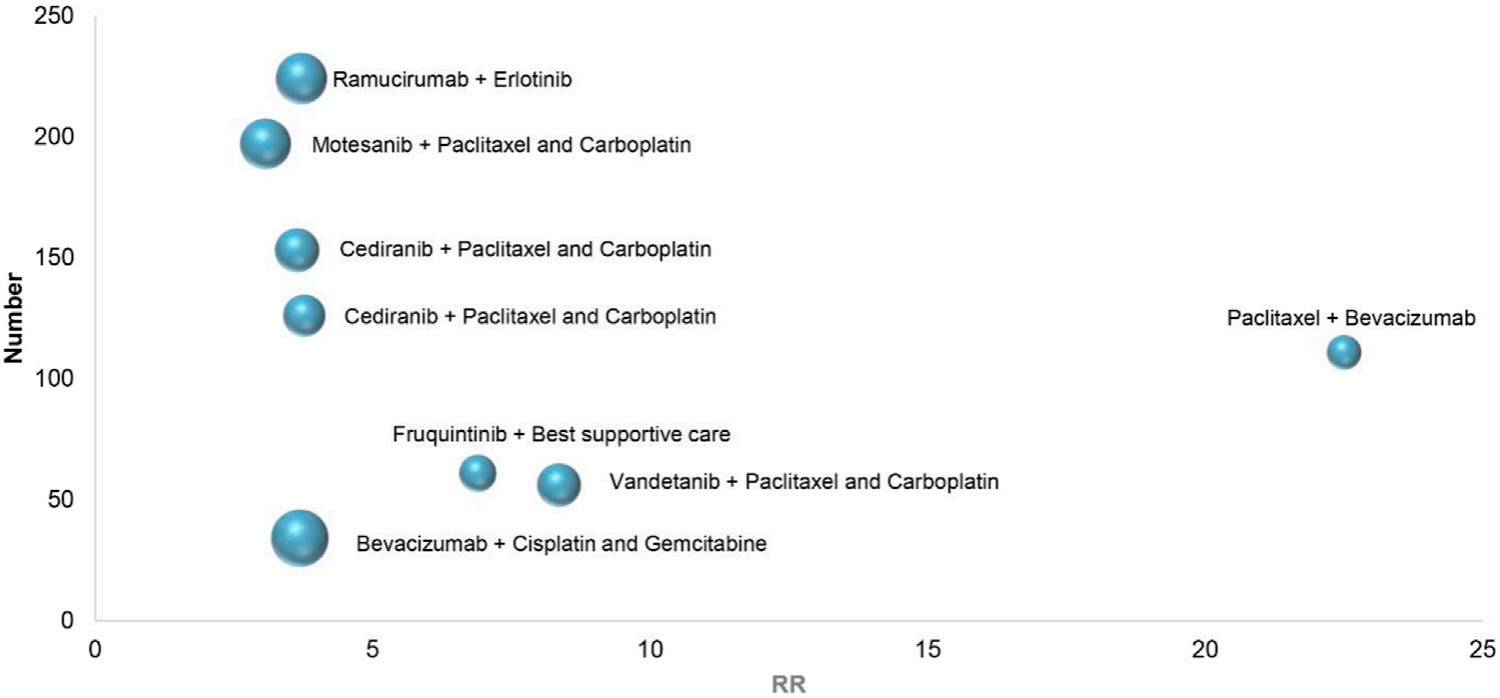
Bubble chart: Non-small cell lung cancer treatment.

In eight RCTs on the treatment of non-small cell lung cancer, combination therapies included vandetanib combined with Paclitaxel and Carboplatin (All-grade: RR 8.36, 95% CI 2.04–32.27; 1–2 grade: RR 6.50, 95% CI 1.55–27.24; 3–4 grade: RR 8.37, 95% CI 0.46–151.74) (Heymach et al., 2008), cediranib combined with Paclitaxel and Carboplatin (All-grade: RR 3.77, 95% CI 1.97–7.23; 1–2 grade: RR 2.36, 95% CI 1.07–5.18; 3–4 grade: RR 9.42, 95% CI 2.24–39.61) (Goss et al., 2010), bevacizumab combined with Cisplatin and Gemcitabine (All-grade: RR 3.69, 95% CI 1.56–8.72; 1–2 grade: RR 3.11, 95% CI 1.28–7.51; 3–4 grade: RR 6.80, 95% CI 0.36–126.76) (Mok et al., 2011), cediranib combined with Paclitaxel and Carboplatin (All-grade: RR 3.64, 95% CI 2.11–6.30; 1–2 grade: RR 3.27, 95% CI 1.73–6.19; 3–4 grade: RR 5.00, 95% CI 1.48–16.92) (Laurie et al., 2014), motesanib combined with Paclitaxel and Carboplatin (All-grade: RR 3.07, 95% CI 2.12–4.46; 1–2 grade: RR 2.24, 95% CI 1.45–3.44; 3–4 grade: RR 8.28, 95% CI 2.98–22.99) (Kubota et al., 2017), fruquintinib combined with Best supportive care (All-grade: RR 6.89, 95% CI 0.95–49.92; 1–2 grade: RR 9.50, 95% CI 0.57–157.94; 3–4 grade: RR 2.46, 95% CI 0.30–20.12) (Lu et al., 2018), ramucirumab combined with erlotinib (All-grade: RR 3.72, 95% CI 2.54–5.45; 1–2 grade: RR 3.21, 95% CI 1.86–5.57; 3–4 grade: RR 4.35, 95% CI 2.39–7.93) (Nakagawa et al., 2019), Paclitaxel combined with bevacizumab (All-grade: RR 22.50, 95% CI 1.39–364.14; 1–2 grade: RR 14.50, 95% CI 0.88–238.66; 3–4 grade: RR 8.50, 95% CI 0.50–144.62) (Cortot et al., 2020). Depending on the above data, Figure 4, in 8 RCTs of non-small cell lung cancer, the highest RR of hypertension caused by Paclitaxel combined with bevacizumab, With the increase of the number of patients, ramucirumab combined with erlotinib has a relatively small and better chance of inducing hypertension in the treatment of non-small cell lung cancer.

**FIGURE 5:**
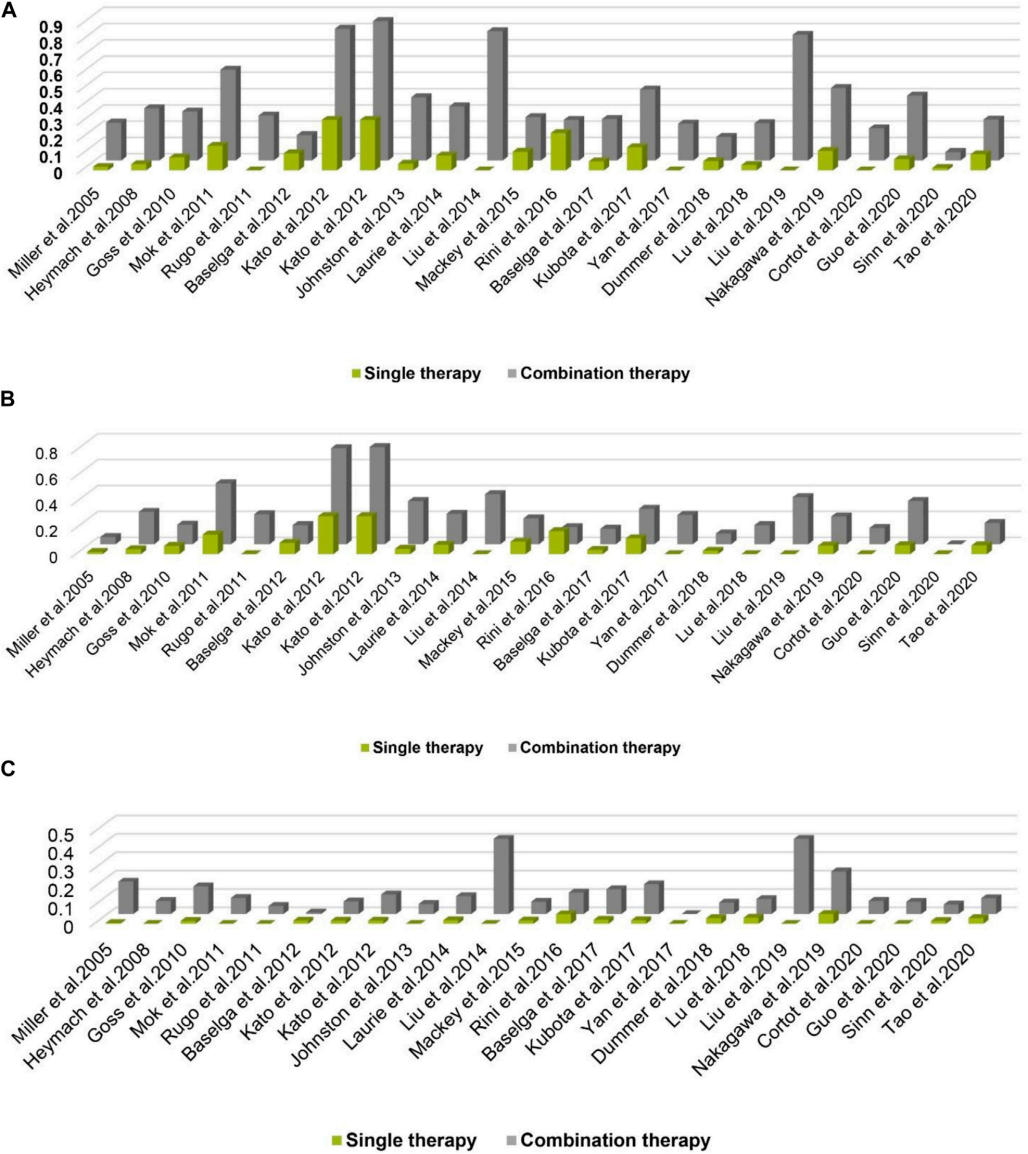
Incidence rate of hypertension.

**FIGURE 6:**
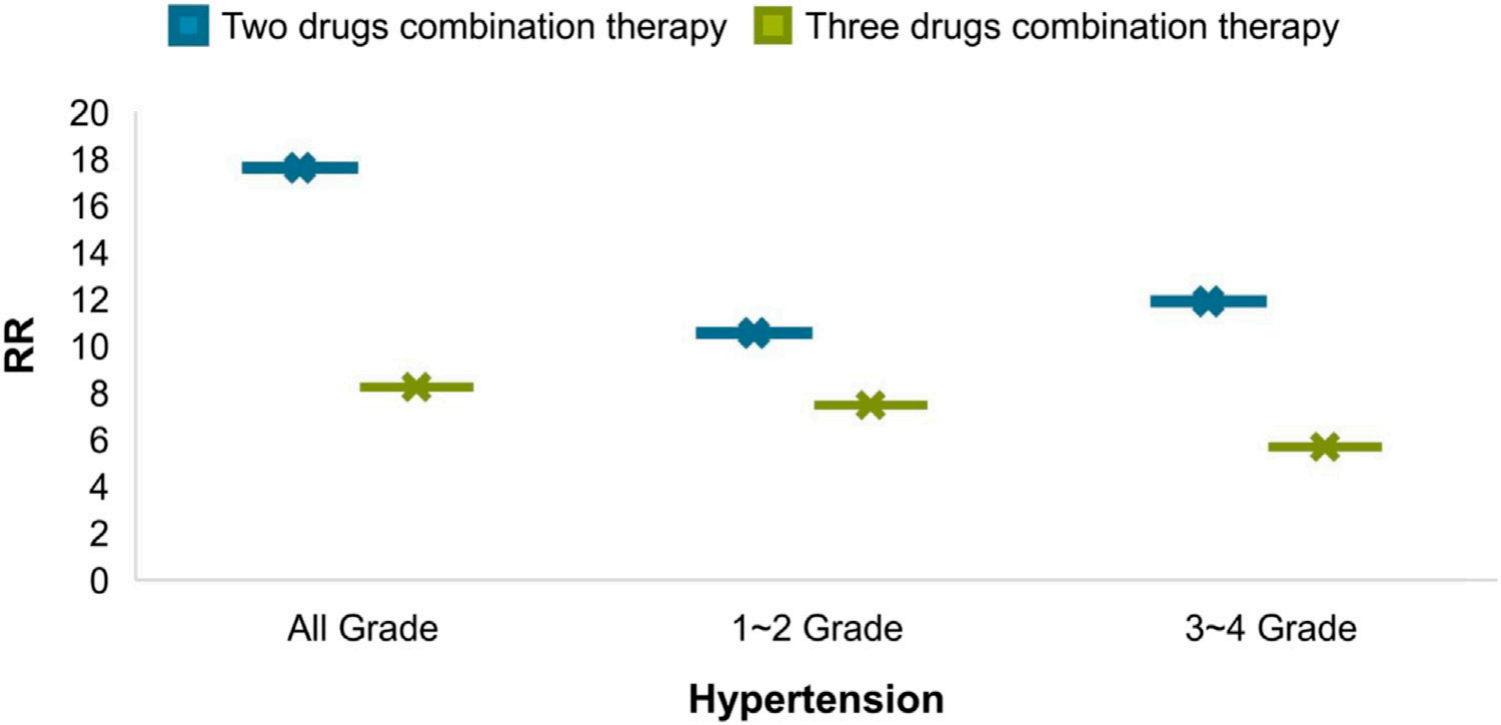
Comparison of RR of hypertension caused by cancer combination therapy regimens.

From an intuitive point of view, the incidence of hypertension caused by combination therapy of cancer is higher than that of single therapy, whether it is at all-grade, 1–2 grade or 3–4 grade hypertension, the results shown in Figure 5A–C. As cancer combination therapy regimens, Figure 6, the result of analyze show that the RR of hypertension caused by two drugs combination therapy is higher than three drugs combination therapy, because there are very few plans of multi-drug (*n* > 3) combination therapy, it is not included as a comparison. For more details of the other schemes, please refer to Table 1.

### Heterogeneity and Bias of Included Studies

**FIGURE 7:**
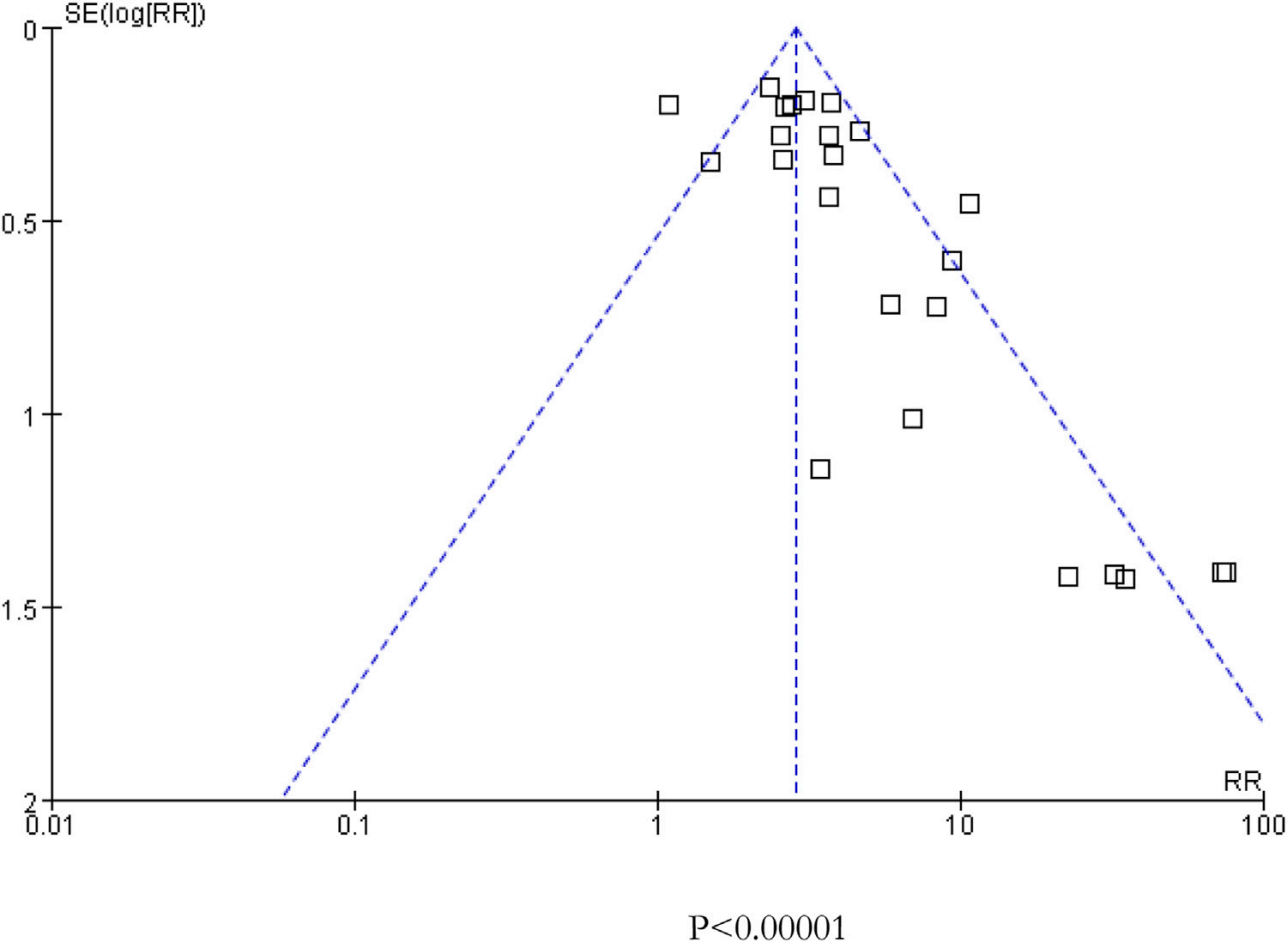
Funnel plot for all included 23 studies included in the meta-analysis.

**FIGURE 8:**
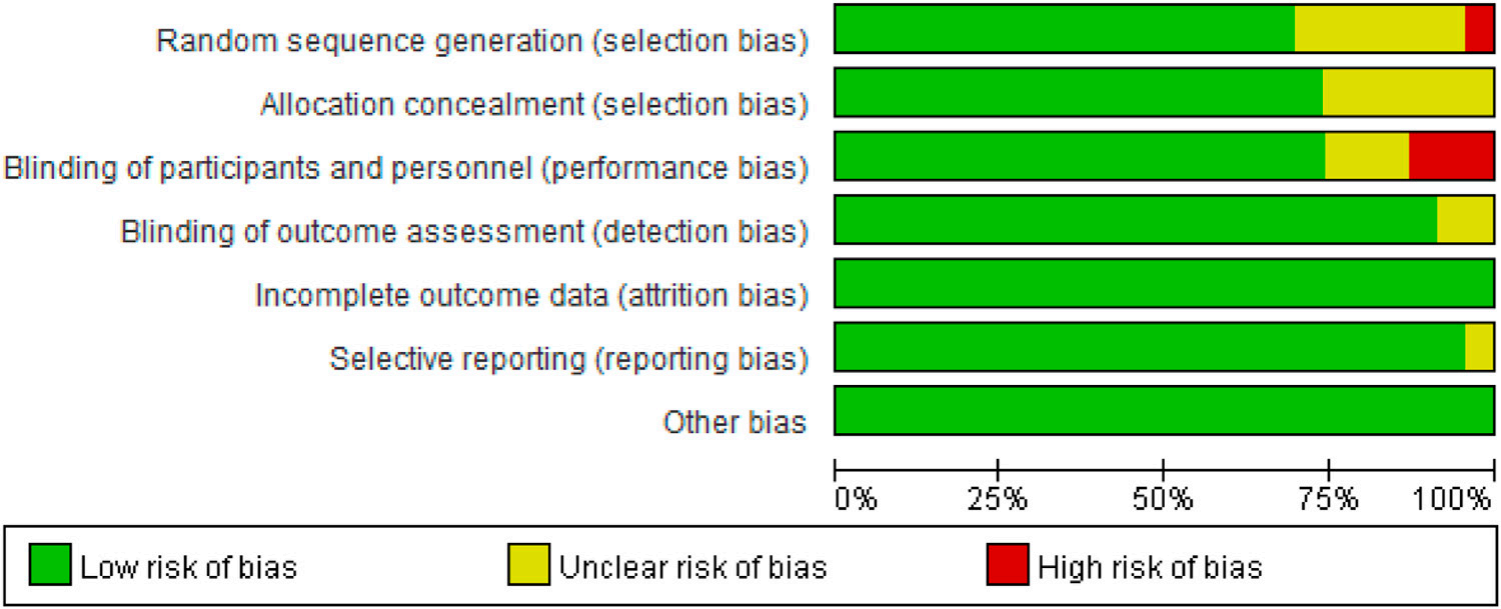
Risk of bias of included randomized controlled trials.

**FIGURE 9:**
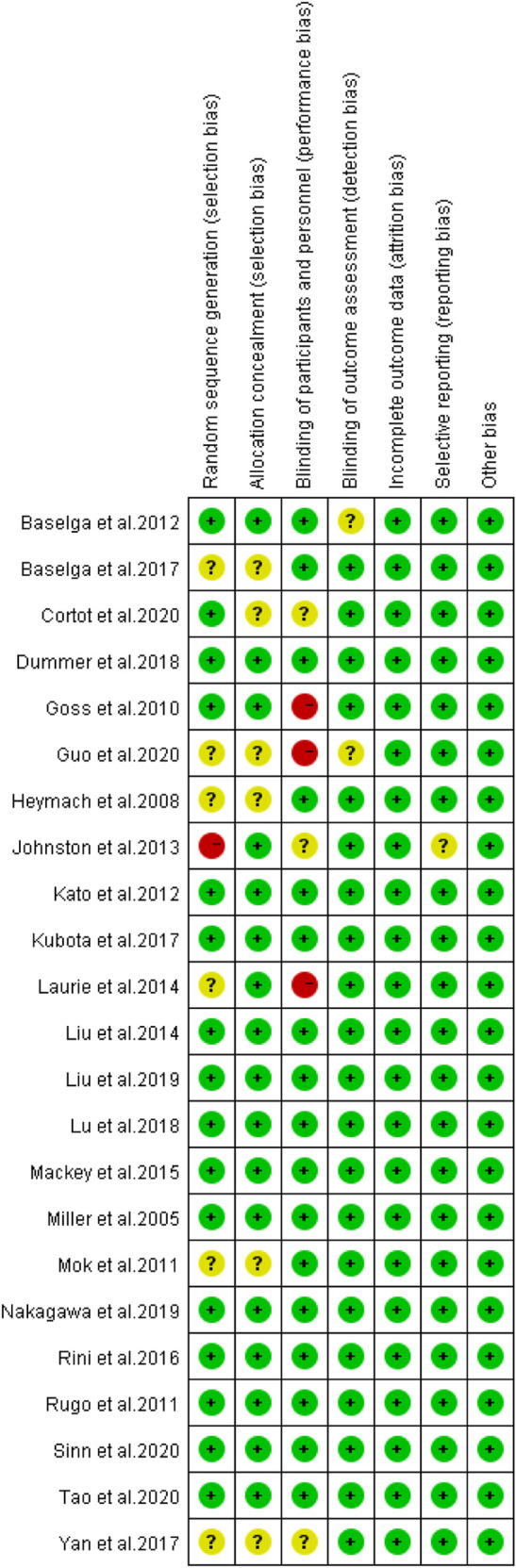
Risk of bias of included randomized controlled trials summary.

As presented in Figure 2, there was moderate heterogeneity in grade 1–2 hypertension (*I* ^*2*^ = 55%, *p* < 0.001), low heterogeneity in grade 3–4 hypertension (*I* ^*2*^ = 5%, *p* = 0.39), and moderate heterogeneity in all grades of hypertension (*I* ^*2*^ = 68%, *p* < 0.001) caused by cancer combination therapy, with the presence of statistical significance. Using the risk-of-bias assessment tool (Higgins et al., 2011), the results of the Cochrane risk-of-bias assessment of the enrolled 23 RCTs are shown in Table 4 and Figures 7–9 showed that the funnel plot indicated evidence of heterogeneities and publication bias in the studies included in the meta-analysis with scatters beyond 95% CI and asymmetry display (*p* < 0.00001).

**TABLE 4:** Risk of bias of included randomized controlled trials.

Author (Year)	Random sequence generation (selection bias)	Allocation concealment (selection bias)	Blinding of participants and personnel (performance bias)	Blinding of outcome assessment (detection bias)	Incomplete outcome data (attrition bias)	Selective reporting (reporting bias)	Other bias
Baselga et al. (2012)	Low	Low	Low	Unclear	Low	Low	Low
Baselga et al. (2017)	Unclear	Unclear	Low	Low	Low	Low	Low
Cortot et al. (2020)	Low	Unclear	Unclear	Low	Low	Low	Low
Dummer et al. (2018)	Low	Low	Low	Low	Low	Low	Low
Goss et al. (2010)	Low	Low	High	Low	Low	Low	Low
Guo et al. (2020)	Unclear	Unclear	High	Unclear	Low	Low	Low
Heymach et al. (2008)	Unclear	Unclear	Low	Low	Low	Low	Low
Johnston et al. (2013)	High	Low	Unclear	Low	Low	Low	Low
Kato et al. (2012)	Low	Low	Low	Low	Low	Low	Low
Kato et al. (2012)	Low	Low	Low	Low	Low	Low	Low
Kubota et al. (2017)	Low	Low	Low	Low	Low	Low	Low
Laurie et al. (2014)	Unclear	Low	High	Low	Low	Low	Low
Liu et al. (2014)	Low	Low	Low	Low	Low	Low	Low
Liu et al. (2019)	Low	Low	Low	Low	Low	Low	Low
Lu et al. (2018)	Low	Low	Low	Low	Low	Low	Low
Mackey et al. (2015)	Low	Low	Low	Low	Low	Low	Low
Miller et al. (2005)	Low	Low	Low	Low	Low	Low	Low
Mok et al. (2011)	Unclear	Unclear	Low	Low	Low	Low	Low
Nakagawa et al. (2019)	Low	Low	Low	Low	Low	Low	Low
Rini et al. (2016)	Low	Low	Low	Low	Low	Low	Low
Rugo et al. (2011)	Low	Low	Low	Low	Low	Low	Low
Sinn et al. (2020)	Low	Low	Low	Low	Low	Low	Low
Tao et al. (2020)	Low	Low	Low	Low	Low	Low	Low
Yan et al. (2017)	Unclear	Unclear	Unclear	Low	Low	Low	Low

## Discussion

To our knowledge, the present meta-analysis for the first time evaluated the potential risk of hypertension in cancer patients treated with combination therapy. As a “silent killer,”, hypertension has been reported to have a doubled prevalence in the past 40 years, with 7.6 million people dying of hypertension annually in the world (Arima et al., 2011). Despite no significant direct influence, long-term hypertension may result in damage of the heart and blood vessels, and cerebral artery vasospasm as well.

In the field of Cardio-Oncology, cancer combination therapy may produce the effective outcome in killing cancer cells and controlling the deterioration of cancer. Nevertheless, there is an inevitable adverse effect of heart disease, especially the occurrence of hypertension. In this regard, there is an urgent need for medical staff to adjust the therapeutic schemes of patients, timely prevent and alleviate side effects during and after cancer treatment, to ensure the life safety of patients.

Current anti-hypertensive therapeutics included Selective α1 adrenoceptor antagonist, non-selective α1 and α2-antagonists, β-adrenoceptor antagonists, angiotensin II receptor blockers, calcium channel blockers, ACE inhibitors, renin inhibitors, direct vasodilators, loop diuretics (Kumar et al., 2020). However, we should pay more attention to the related complications which they are accompanied, such as organ damage, hypotension and so on (Kumar et al., 2020).

In our meta-analysis, based on the collection of all relevant data from retrospective clinical trials, the final objects of study were a total of 23 clinical trials involving 6,241 patients. The combination therapy of cancer patients resulted in a higher risk of developing hypertension (All-grade: RR 2.85, 95% CI 2.52–3.22; 1–2 grade: RR 2.43, 95% CI 2.10–2.81; 3–4 grade: RR 4.37, 95% CI 3.33–5.72). According to the results, the risk of grade 3–4 hypertension induced by cancer combination therapy was higher than that of the control group with or without placebo therapy.

**FIGURE 10:**
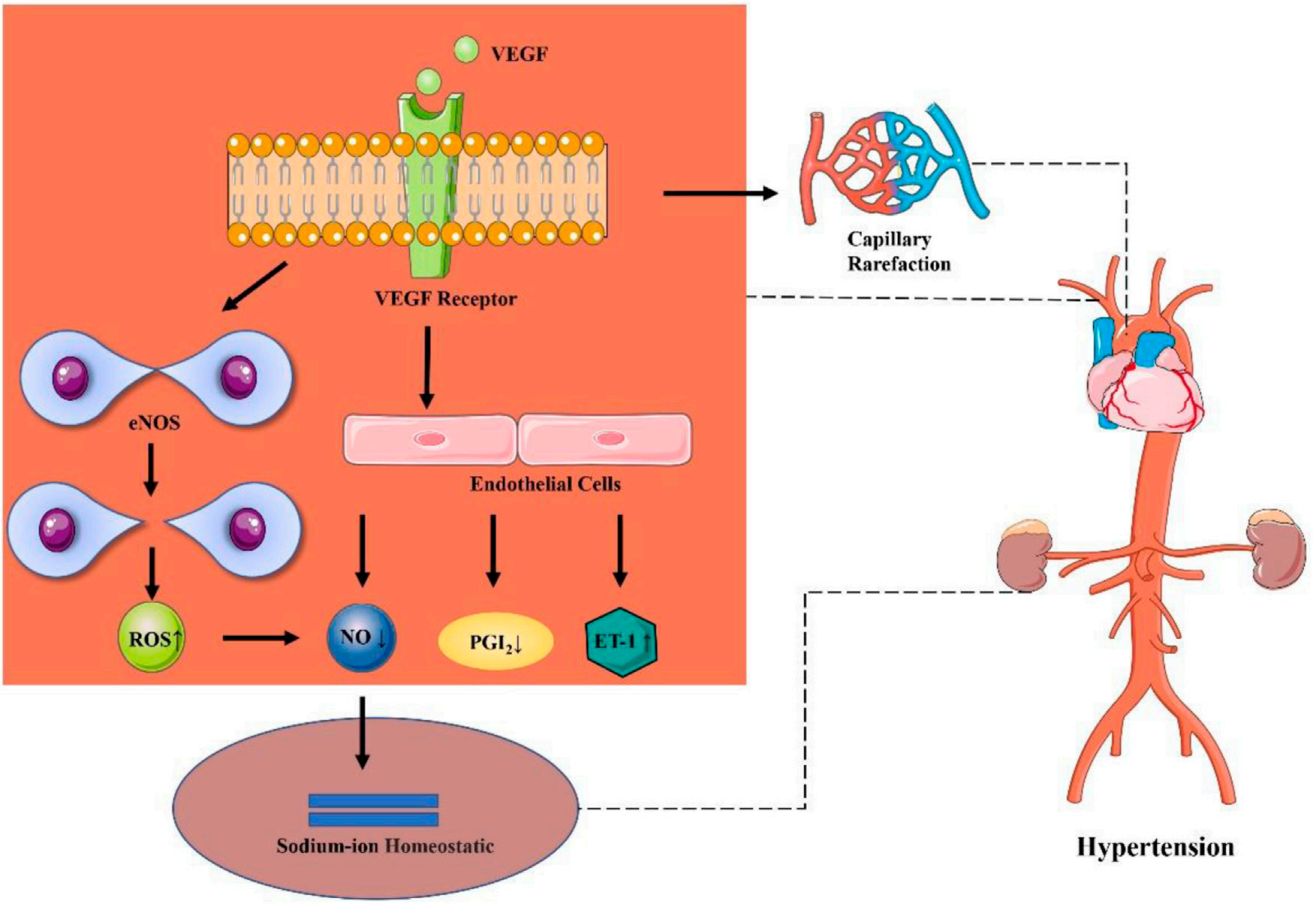
Possible mechanism of hypertension caused by cancer therapies.

There may exist different mechanisms of increase in blood pressure under different anti-cancer therapeutic schemes. The mechanism of elevated blood pressure by using anti-cancer drugs may exhibit a direct association with its anti-cancer mechanism. The mechanism of hypertension induced by cancer combination therapy may be explained by the following reasons. To be specific, monoclonal antibodies (for example, bevacizumab) may reduce the number of capillaries in microcirculation, competitively inhibit the binding of EGFR with other ligands, and block the interaction between VEGF and endothelial cell surface receptors, resulting in inhibit the signal pathway of VEGF, reduce the activity of endothelial nitric oxide synthase and the production of NO and PGI_2_ by vascular endothelial cells, decrease vascular permeability and vasodilation, increased peripheral vascular resistance and blood flow, and finally lead to hypertension (Chen et al., 2011; Mayer et al., 2011; Mourad and Levy, 2011; Campia et al., 2019). Besides, it has been reported that reducing the activity of eNOS will lead to expression of uncoupling protein of eNOS, produces a large amount of reactive oxygen species and then decrease the level of NO (Kumar et al., 2020). Meanwhile, NO is involved in maintaining the steady state of sodium ions and participating in tubuloglomerular feedback to regulate renal blood flow and glomerular filtration, which can increase systemic blood pressure (Lankhorst et al., 2017). Another possible mechanism of hypertension caused by inhibiting other VEGF pathways is that angiogenesis inhibitors may reduce the number of blood vessels and lead to hypertension owing to the thinning of peripheral microvessels (Aparicio-Gallego et al., 2011). In addition, additional research also reveals that the increase in blood pressure may be related to the inhibition of VEGFR-2 (Kamba and McDonald, 2007). Also, Small molecular targeted drugs (such as sunitinib) can upregulate endothelin-1, increase salt sensitivity, and further increase in blood pressure owing to thrombotic glomerular injury (Kidoguchi et al., 2021). In addition, some novel targeted drugs (e.g., brutinib) may increase the risk of hypertension by inhibiting PI3K/Akt or reducing the level of NO (Dickerson et al., 2019). (Figure 10)

With respect to the above, there is necessary to adopt targeted treatment of hypertension. Before the treatment of cancer patients, it is recommended to adopt a comprehensive risk assessment, including blood pressure measurement and examination of known risk factors. For cancer patients with existed cardiovascular diseases, it is necessary to consider carefully whether to use anti-cancer drugs that may lead to cardiotoxicity or not. In the field of Cardio-Oncology, further consideration of the overall health status of patients is required for doctors to make a prudent decision in patients with a high risk of hypertension and those with hypertension prior to the use of anti-cancer drugs. Moreover, in case of poor control of cancer development by monotherapy, the better therapeutic outcome may be produced by combination therapy, However, it should be noted that combination therapy may also lead to a higher risk of hypertension.

So far, there is still no systematic analysis of hypertension caused by cancer combination therapy. Data in our study fully supports that cancer combination therapy has a high risk of inducing hypertension. Findings in this meta-analysis suggest that much attention shall be paid constantly to the adverse reactions of combined use of drugs, with in-time prevention required simultaneously. However, there are limitations in this study. For example, due to the absence of experimental data, relevant experiments are needed in the future to fully clarify the pathophysiological basis of hypertension caused by the combination of drugs and to increase the credibility of the results of our study.

## Conclusion

The accuracy of meta-analysis research is high, but there is also a certain degree of publication bias, and risk of bias is low. It is worth mentioned that the reliability of meta-analysis results as well as the suitability in clinical practice might still requires critical thinking and objective judgments.

To sum up, the present meta-analysis carries out a comprehensive analysis on the risk of patients suffering from hypertension in the process of multiple cancer combination therapies. Findings in our study support that the risk of hypertension may increase significantly in cancer patients with multiple cancer combination therapies. The outcomes of this meta-analysis may provide a reference value for clinical practice and may supply insights in reducing the incidence of hypertension caused by cancer combined treatment.
